# Daphnetin Ameliorates Neuropathic Pain via Regulation of Microglial Responses and Glycerophospholipid Metabolism in the Spinal Cord

**DOI:** 10.3390/ph17060789

**Published:** 2024-06-16

**Authors:** Wulin Liang, Tianrui Zhang, Mingqian Zhang, Jiahui Gao, Rikang Huang, Xiyan Huang, Jianhua Chen, Lu Cheng, Liyuan Zhang, Zhishan Huang, Qiling Tan, Zhanhong Jia, Shuofeng Zhang

**Affiliations:** 1 School of Chinese Materia Medica, Beijing University of Chinese Medicine, Beijing 102488, China; 2 Shanxi Provincial Key Laboratory of Drug Toxicology and Preclinical Research of Radiopharmaceuticals, Key Laboratory of Radiotoxicology and Preclinical Evaluation of Radiopharmaceuticals in China, National Atomic Energy Agency Nuclear Technology Research and Development Center, Institute of Radiology and Environmental Medicine, China Institute For Radiation Protection, Taiyuan 030006, China

**Keywords:** daphnetin, neuropathic pain, microglia, purinergic P2X_4_, P38 mitogen-activated protein kinases, glycerophospholipid metabolism

## Abstract

Neuropathic pain (NP) is a common type of chronic pain caused by a lesion or disease of the somatosensory nervous system. This condition imposes a considerable economic burden on society and patients. Daphnetin (DAP) is a natural product isolated from a Chinese medicinal herb with various pharmacological activities, such as anti-inflammatory and analgesic properties. However, the underlying mechanisms of these effects are not fully understood. In the present study, we aimed to investigate DAP’s anti-inflammatory and analgesic effects and explore the underlying mechanisms of action. The NP model was established as chronic constrictive injury (CCI) of the sciatic nerve, and pain sensitivity was evaluated by measuring the mechanical withdrawal threshold (MWT) and thermal withdrawal threshold (TWT). The activation of microglia in the spinal dorsal horn was measured via immunofluorescence staining. Protein levels were measured using a western blot assay. Using a mass-spectrometry proteomics platform and an LC-MS/MS-based metabolomics platform, proteins and metabolites in spinal cord tissues were extracted and analyzed. DAP treatment ameliorated the MWT and TWT in CCI rats. The expression of IL-1β, IL-6, and TNF-α was inhibited by DAP treatment in the spinal cords of CCI rats. Moreover, the activation of microglia was suppressed after DAP treatment. The elevation in the levels of P2X_4_, IRF8, IRF5, BDNF, and p-P38/P38 in the spinal cord caused by CCI was inhibited by DAP. Proteomics and metabolomics results indicated that DAP ameliorated the imbalance of glycerophospholipid metabolism in the spinal cords of CCI rats. DAP can potentially ameliorate NP by regulating microglial responses and glycerophospholipid metabolism in the CCI model. This study provides a pharmacological justification for using DAP in the management of NP.

## 1. Introduction

Neuropathic pain (NP) is defined as “pain caused by primary lesions, dysfunction or transient disturbance of the peripheral or central nervous system” [1]. In most cases, NP is caused by peripheral or central nerve lesions. An epidemiological study has shown that the prevalence of NP within the general population could be as high as 7% to 8%, accounting for 20% to 25% of patients with chronic pain [2]. Continuous stimulation, like tissue or nerve injury, can reshape nociceptors and engender pain sensitization, hyperalgesia, and allodynia [3]. Central sensitization of the spinal cord and supraspinal cord remains a hot research topic in NP pathogenesis [4].

In recent years, NP and the abnormal excitation of neurons have been studied in depth. Nonneural cells of the systema nervosum, such as monocytes, macrophages, T cells, and glial cells, play a vital role in the study of NP [5]. In 2003, P2X purine receptors 4 (P2X_4_) and P38 mitogen-activated protein kinase (P38 MAPK) were found to be microglia-specific molecules that are upregulated and activated after NP, and the causative role of spinal microglia in NP was determined for the first time [6]. Deleting the P2X_4_ receptors and conducting targeted intervention have been reported to scale back hyperalgesia in animal pain models [7]. Therefore, the P2X_4_/P38 MAPK pathway in microglia might be a therapeutic target for NP.

**Figure 1:**
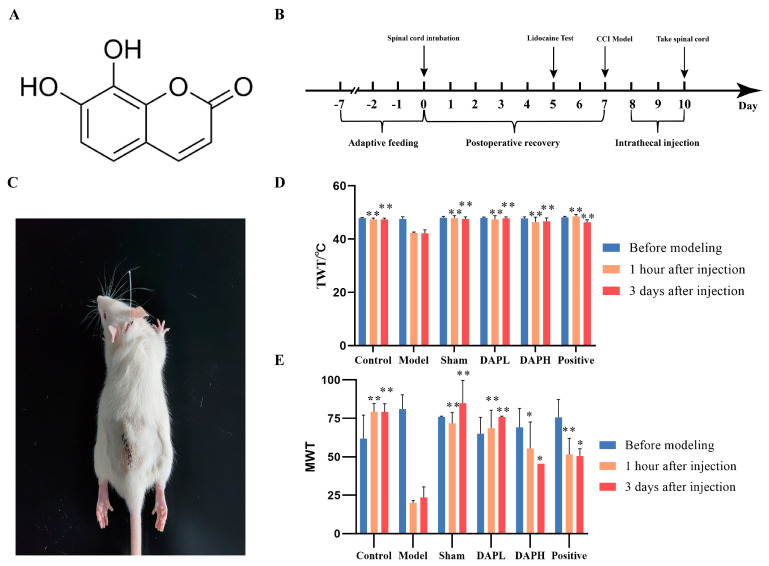
Effect of DAP on pain sensation in CCI rats. (**A**) Structural formula of DAP. (**B**) Experimental flowchart. (**C**) Spinal cord subdural intubation in rats. The rats were intubated at the L4−L5 spinal cord in the subdural cavity, as shown in the figure. After the injection of lidocaine hydrochloride, they immediately became paralyzed. (**D**) Change in TWT in CCI rats at different administration times (*n* = 6). (**E**) Change in MWT in CCI rats at different administration times (*n* = 6). * *p* < 0.05, ** *p* < 0.01 vs. model group.

Daphnetin (DAP) (7, 8-dihydroxy coumarin) is a natural coumarin compound isolated from the stem and root bark of *Daphne giraldii* Nitsche, a plant of the *Daphne* family. Its structural formula is shown in Figure 1A. DAP exhibits a wide range of biological activities, including antioxidant, analgesic, and anti-inflammatory effects [8]. It was found that DAP inhibited LPS-induced production of the inflammatory mediators IL-1β, TNF-α, and nitric oxide in BV2 cells [9]. In animal studies, DAP can treat autoimmune diseases, including experimental autoimmune encephalomyelitis and rheumatoid arthritis, which is related to its analgesic function [10,11]. Previous studies have shown that the intraperitoneal injection and gavage of DAP ameliorated inflammatory pain induced by complete Freund’s adjuvant in mice and NP induced by peripheral nerve injury in rats, and the mechanism of action was related to the inhibition of spinal glial-cell activation [12,13]. These studies demonstrate that the spinal cord may be an effective target site for DAP analgesia. We used intrathecal injection for the administration of DAP to further clarify the effects of DAP in ameliorating NP at the spinal cord level and the mechanism of action. Our previous study found that DAP could inhibit the activation of spinal microglia in the TNF-α-induced NP rat model, which has profound implications for this study [14]. Therefore, during this study, we aimed to determine whether DAP can treat CCI-induced NP through spinal cord administration and explore the effects of DAP on the P2X_4_/P38 pathway of microglia under central sensitization conditions. In addition, we evaluated the effects of DAP on protein expression and metabolite profiles of the spinal cord using proteomics and metabolomics analysis to further reveal the mechanism underlying the anti-nociceptive effect of DAP.

## 2. Results

### 2.1. DAP Improved NP in CCI Rats

In this study, we constructed an intrathecal injection mode before creating the chronic constrictive injury (CCI) pain model, as shown in Figure 1C. This method of administration can be repeated through an EP-10 catheter at the L4−L5 position without damaging the spinal cord. We established the CCI model 7 days after subdural intubation, as shown in Figure 1B. We measured the thermal withdrawal threshold (TWT) and mechanical withdrawal threshold (MWT) of CCI rats before modeling, 1 h after administration, and 3 days after administration. Before modeling, there was no significant difference in the TWT and MWT in between groups (*p* > 0.05), which proved that subdural catheterization did not cause pain sensitization in rats. One hour after the administration, compared with the sham group, the TWT and MWT of the model group were significantly reduced (*p* < 0.05), which proved that the CCI model had been successfully established. At this time, the rats showed thermal and mechanical allodynia. Similarly, there was no significant difference in the TWT and MWT between the sham group and control group, which further confirmed that subdural catheterization would not induce pain sensitization in rats. The positive, DAP high−dose (DAPH), and DAP low−dose (DAPL) groups exhibited a significant increase in the TWT and MWT compared with the model group (*p* < 0.05). After 3 days of continuous administration, there was still a significant difference in the TWT and MWT between the model and sham groups (*p* < 0.05), proving that pain sensitization persisted (Figure 1D,E).

### 2.2. DAP Inhibited the Activation of Microglia and the Expression of Inflammatory Factors in the Spinal Cords of CCI Rats

**Figure 2:**
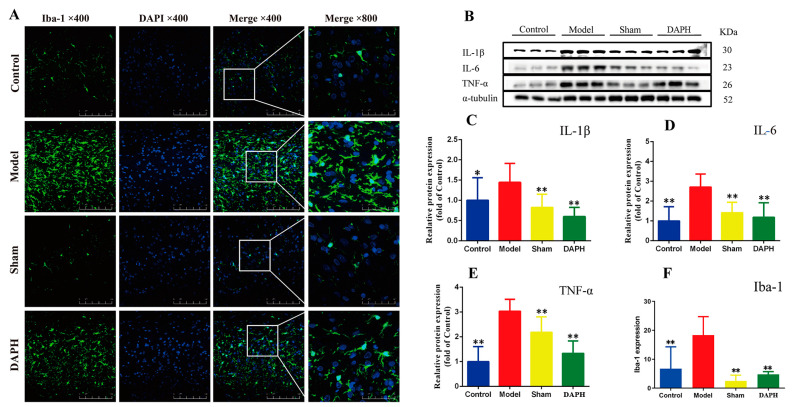
Effect of DAP on microglia activation and expression of inflammatory factors in the spinal cords of CCI rats. (**A**) The rat spinal cord microglia marker Iba-1 (green) was stained with immunofluorescence. The scale bar is 20 μm (*n* = 3). (**B**–**E**) Expression of IL-1β, IL-6, and TNF-α proteins in the spinal cord (*n* = 3). (**F**) Fluorescence intensity of Iba-1 in immunofluorescence. * *p* < 0.05, ** *p* < 0.01 vs. model group.

It has been found that neuroinflammation caused by microglia activation plays a vital role in the pathogenesis and maintenance of NP [15]. Microglia are involved in the initial signal regulation of neuronal activation, which is commonly accompanied by high expression of microglia in the spinal dorsal horn of the NP model [16]. In this experiment, we observed high Iba-1 expression in the spinal dorsal horn of CCI rats, which proved that the microglia in the spinal dorsal horn of CCI rats were extensively activated and participated in the regulation of NP. After 3 days of intrathecal injection of DAP, the expression of Iba-1 in the spinal cords of CCI rats was considerably downregulated and the activation of microglia was significantly inhibited (Figure 2A,F).

Peripheral nerve injury induces macrophage aggregation and cytokine production, leading to neuronal sensitization and inflammation in the spinal dorsal horn [17]. The CCI model can induce microglia to release TNF-α, IL-1β, and IL-6 [18]. To explore whether DAP can regulate related inflammatory factors, we analyzed the expression of inflammatory factors in the spinal cords of CCI rats. The results showed that expression of TNF-α, IL-1β, and IL-6 in the spinal cords of CCI rats was significantly increased, as is consistent with reports in the literature. After 3 days of intrathecal injection of DAP, the expression of inflammatory factors in the spinal cords of CCI rats was significantly inhibited, which may be associated with the inhibition of microglia activation by DAP (Figure 2B–E).

### 2.3. DAP Regulated P2X_4_ Receptors in Spinal Microglia of CCI Rats by Interfering with IRF8 Protein

**Figure 3:**
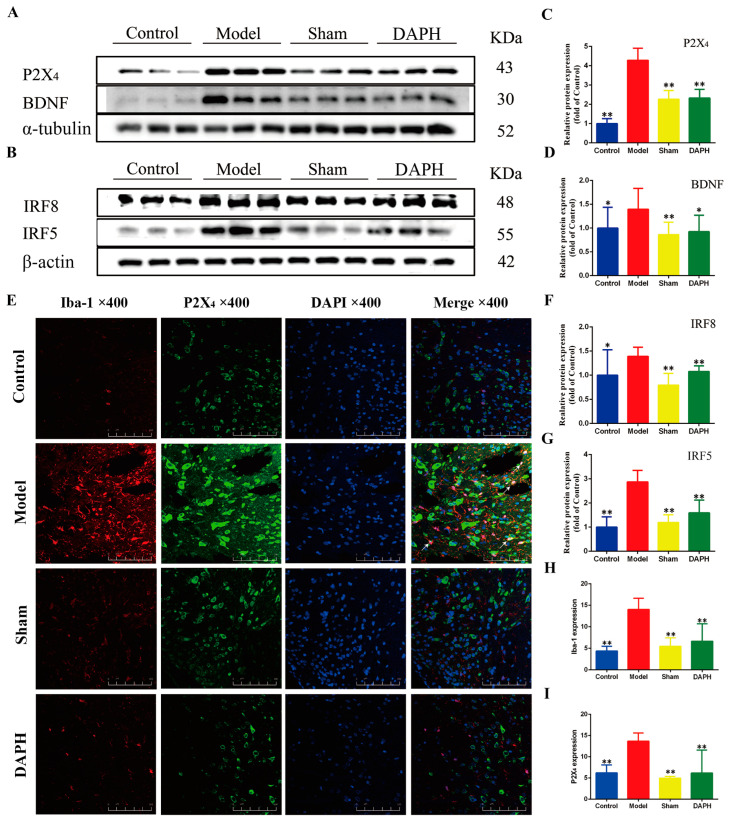
Effect of DAP on P2X_4_ receptors in spinal microglia in CCI rats. (**A**–**D**,**F**,**G**) Expression of IRF8, IRF5, P2X_4_, and BDNF proteins in spinal cord samples (*n* = 3). (**E**,**H**,**I**) Expression of Iba-1 (red) and P2X_4_ (green) receptors in the spinal dorsal horn, as determined by immunofluorescence double staining. The scale bar is 20 μm (*n* = 3). * *p* < 0.05, ** *p* < 0.01 vs. model group.

The P2 purinergic receptor family on the surface of microglia is essential for microglial activation [19]. P2X_4_ and P2X_7_ in the P2X family allow the transfer of organic macromolecules between cells, which can lead to hyperalgesia [20]. In this experiment, we used w blotting to analyze the expression of P2X_4_ protein in the L4−L5 spinal cords of CCI rats and found that it was significantly increased. These findings, combined with double immunofluorescence staining, revealed that P2X_4_ protein was highly expressed in activated microglia in the spinal cords of CCI rats, which is consistent with the trend in protein expression seen in the western blot results. After intrathecal injection of DAP, we observed significant inhibition of the upregulation of P2X_4_ protein in the western blot and immunofluorescence results (Figure 3A,C,E,H,I).

We explored the upstream and downstream target proteins of DAP to further explore its mechanism of intervention in the expression of P2X_4_ protein. Masuda [21] recently found that the expression of interferon regulatory factor (IRF) 8 was selectively upregulated in microglia after nerve injury. It was also found that IRF5 was the target of IRF8. After nerve injury, microglia in the ipsilateral spinal dorsal horn also selectively expressed IRF5 [22]. Accordingly, we determined the relative expression of IRF8 and IRF5 by western blotting. We found that the expression of IRF8 and IRF5 was significantly upregulated in the spinal cords of CCI rats. The upregulation of IRF8 and IRF5 expression was inhibited after intrathecal injection of DAP (Figure 3B,F,G).

Peripheral nerve injury stimulates microglia activation through P2X_4_, which induces microglia to release brain-derived neurotrophic factor (BDNF) [23]. BDNF can act on nociceptive projection neurons in layer I of the spinal cord, and this action can lead to abnormal neuronal excitation and mechanical pain hypersensitivity (allodynia) [24]. We determined the expression of BDNF protein in the spinal cord, and the results are shown in Figure 3A,D. The expression of BDNF protein in the model group increased significantly compared with that in the sham group (*p* < 0.01). After intrathecal injection of DAP, the level of BDNF protein decreased significantly compared with that in the model group (*p* < 0.01). The above experimental results prove that DAP regulates the P2X_4_ receptors in the spinal cords of CCI rats by interfering with the IRF8/IRF5/P2X_4_ axis, thereby inhibiting microglia activation and consequently attenuating NP.

### 2.4. DAP Inhibited the Activation of the MAPK Pathway in Spinal Microglia of CCI Rats by Interfering with P2X_4_ Receptors

**Figure 4:**
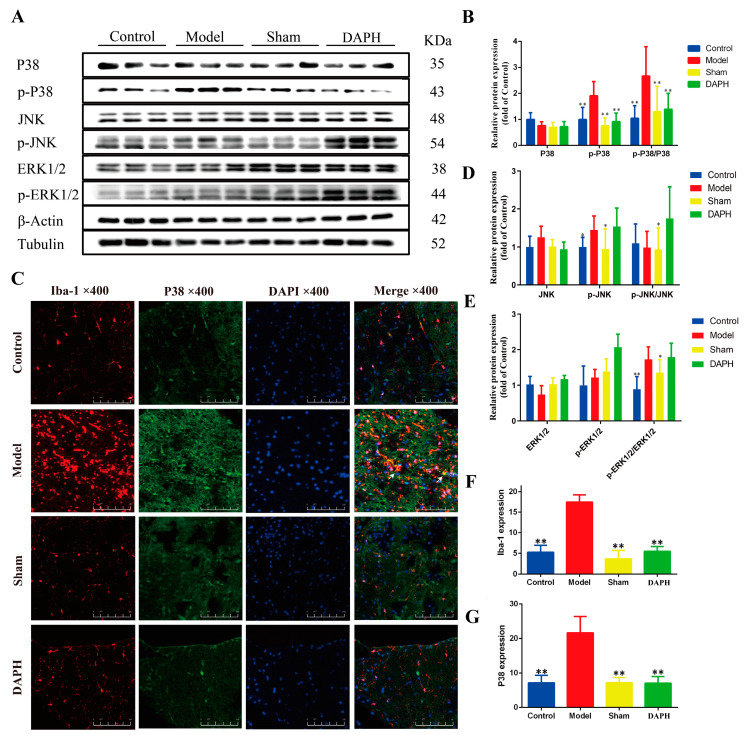
Effect of DAP on MAPK-pathway activation in the spinal microglia of CCI rats. (**A**,**B**,**D**,**E**) The expression of P38/p-P38, JNK/p-JNK, and ERK1/2/p-ERK1/2 proteins in the spinal cord (*n* = 3). (**C**,**F**,**G**) The expression of Iba-1 and P38 in the spinal dorsal horn in immunofluorescence double staining. The scale bar is 20 μm (*n* = 3). * *p* < 0.05, ** *p* < 0.01 vs. model group.

MAPKs are evolutionarily conserved intracellular signaling molecules that play a crucial role in regulating gene expression, promoting disease development, and maintaining pain [25]. The MAPK family is divided into three main members: extracellular signal-regulated kinase (ERK), c-Jun N-terminal kinase (JNK), and P38 MAPK [26]. P38 phosphorylation is a common pathway after the activation of cell-surface receptors on microglia [26]. JNK and ERK phosphorylation contribute to the induction and maintenance of NP [27]. We measured P38, JNK, ERK1/2, and their phosphorylated proteins via western blotting and calculated the ratio of phosphorylated protein to total protein to measure the changes in MAPK-pathway-related proteins. The results showed that the protein expression ratios p-P38/P38, p-JNK/JNK, and p-ERK1/2/ERK1/2 in the model group were significantly higher (*p* < 0.05) than those of the sham group, indicating the activation of the MAPK pathway in spinal microglia, which is related to the overexpression of inflammatory factors. After intrathecal injection of DAP, it was found that the protein expression ratio p-P38/P38 decreased significantly compared with that in the model group (*p* < 0.01), while the expression of p-JNK/JNK and p-ERK1/2/ERK1/2 proteins did not change significantly (Figure 4A,B,D,E).

According to the above experimental results, we stained the L4–L5 spinal cords of CCI rats with Iba-1 and P38 MAPK via immunofluorescence double staining. Through fluorescence-intensity analysis, it was found that the fluorescence intensity of Iba-1 and P38 in the spinal dorsal horn of the model group increased (compared with the sham group, *p* < 0.01). The fluorescence of Iba-1 and P38 considerably overlapped, which further verified that P38 protein was expressed in microglia after MAPK-pathway activation. The fluorescence intensity of P38 in the DAPH group was significantly lower than that in the model group (*p* < 0.01), and the fluorescence of Iba-1 and P38 did not overlap. The fluorescence intensity was consistent with the western blot results for protein expression. The fluorescence intensities of Iba-1 and P38 in the sham group did not increase significantly (compared with the control group, *p* > 0.05) (Figure 4C,F,G).

### 2.5. DAP Induced Changes in Spinal Cord Proteomics Profile in CCI Rats

**Figure 5:**
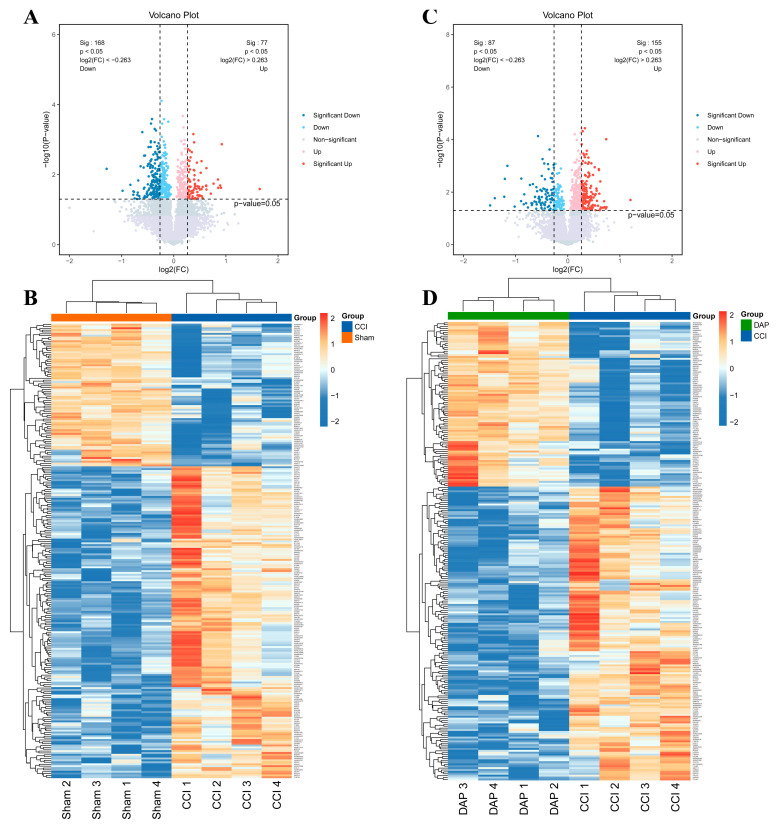
Analysis of DEP expression in the spinal cord samples among the sham, CCI, and DAP groups. (**A**,**B**) The volcano plot and clustering heat map of the DEPs between the CCI and sham groups. (**C**,**D**) The volcano plot and clustering heat map of the DEPs between the DAP and CCI groups.

TMT-based quantitative proteomics was performed to reveal the effects of DAP on CCI-induced abnormal expression of proteins in the spinal cord. A total of 6638 proteins were identified. Based on the filtering criteria of fold change (FC) ≥ 1.2 or FC ≤ 1/1.2 and *p* < 0.05, 245 differentially expressed proteins (DEPs) were screened in the spinal cord samples from the CCI group, including 168 upregulated proteins and 77 downregulated proteins (Figure 5A,B). A total of 242 DEPs were screened out in the DAP group, including 87 upregulated proteins and 155 downregulated proteins (Figure 5C,D).

**Figure 6:**
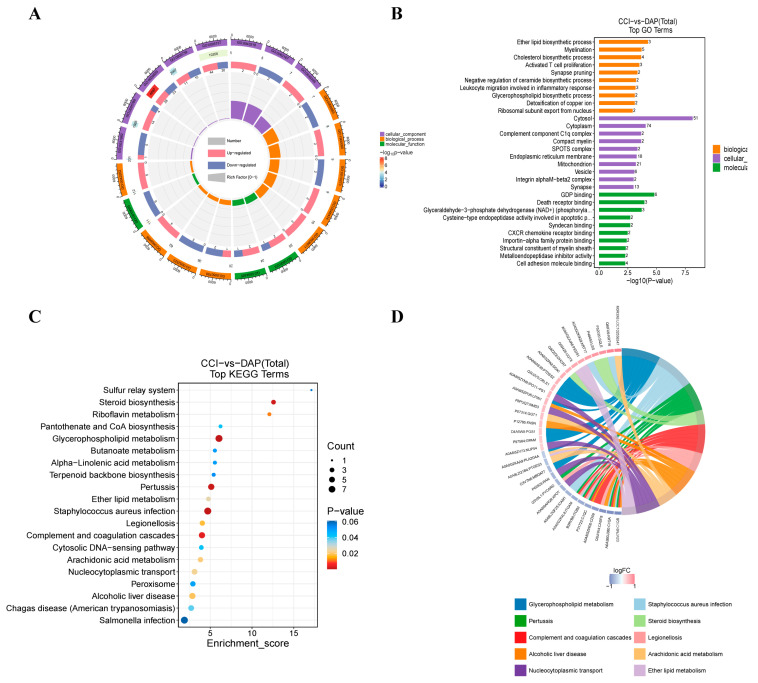
GO and KEGG pathway enrichment analysis of the DEPs between the DAP and CCI groups. (**A**,**B**) GO enrichment analysis of the DEPs between the DAP and CCI groups. (**C**,**D**) KEGG pathway enrichment analysis of the DEPs between the DAP and CCI groups.

GO annotation of the DEPs between the CCI and DAP groups was performed with three categories: cellular components, biological processes, and molecular functions. The top five significantly enriched biological processes were ether lipid biosynthesis, myelination, cholesterol biosynthesis process, activated T-cell proliferation, and synapse pruning (Figure 6A,B). The top five significantly enriched cellular components were cytosol, cytoplasm, the complement component C1q complex, compact myelin, and the SPOTS complex (Figure 6A,B). The top five significantly enriched molecular functions were GDP binding, death-receptor binding, glyceraldehyde-3-phosphate dehydrogenase (NAD+) (phosphorylating) activity, cysteine-type endopeptidase activity involved in the apoptotic process, and syndecan binding (Figure 6A,B). Then, of the DEPs between the CCI and DAP groups was performed to explore the mechanisms underlying DAP’s analgesic effects. The results showed that the DEPs were primarily involved in multiple pathways related to metabolism and inflammation, including steroid biosynthesis, riboflavin metabolism, pantothenate and CoA biosynthesis, glycerophospholipid metabolism, butanoate metabolism, alpha-linolenic acid metabolism, terpenoid-backbone biosynthesis, ether lipid metabolism, complement and coagulation cascades, arachidonic acid metabolism, and peroxisome function (Figure 6C,D).

### 2.6. DAP Induced Changes in Spinal Cord Metabolomics Profile in CCI Rats

**Figure 7:**
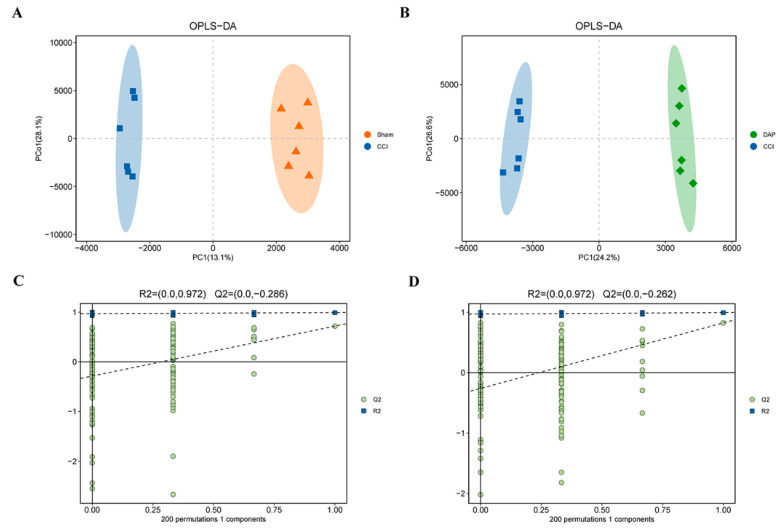
OPLS-DA score plots and permutation tests of spinal cord samples in the sham, CCI, and DAP groups. (**A**,**C**) OPLS-DA score plots and permutation tests in spinal cord samples between the sham and CCI groups. (**B**,**D**) OPLS-DA score plots and permutation tests between spinal cord samples from the DAP and CCI groups.

An untargeted metabolomics study of the spinal cord was performed on the sham, CCI, and DAP groups to reveal the effects of DAP on CCI-induced spinal cord metabolic dysfunction. A total of 4943 metabolites were identified, including 2484 in the positive-ion mode and 2459 in the negative-ion mode. Multidimensional statistical analysis was carried out using orthogonal partial least squares discriminant analysis (OPLS-DA). An explicit separation was shown between the CCI and sham groups and between the DAP and CCI groups in the OPLS-DA model (Figure 7A,B), suggesting that DAP could partly improve CCI-induced spinal cord metabolic dysfunction. Permutation tests showed that the model had good robustness without overfitting (Figure 7C,D).

**Figure 8:**
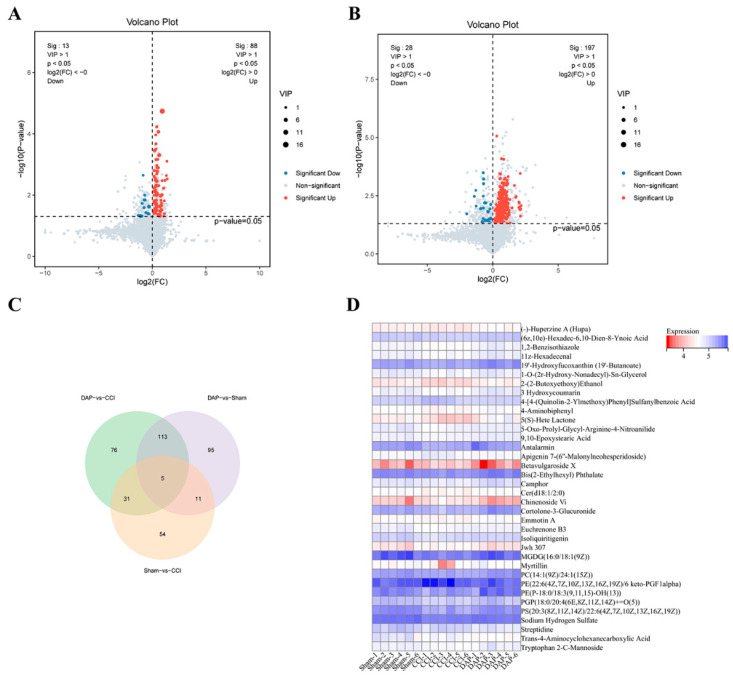
Expression analysis of DEMs in the spinal cord samples among the sham, CCI, and DAP groups. (**A**) Volcano plot of the DEPs between the CCI and sham groups. (**B**) Volcano plot of the DEPs between the CCI and DAP groups. (**C**) Venn diagram of DEMs in the CCI vs. sham and DAP vs. CCI groups. (**D**) Heat map of 36 DEPs between the CCI vs. sham and DAP vs. CCI groups.

In the OPLS-DA model, variable importance in projection (VIP) > 1 and *p* < 0.05 were used to identify significantly differential metabolites (DEMs). In total, 101 DEMs were screened out in the spinal cord samples of the CCI group, including 88 upregulated metabolites and 13 downregulated metabolites (Figure 8A). A total of 225 DEMs were found in the DAP group, including 197 upregulated metabolites and 28 downregulated metabolites (Figure 8B). A total of 36 common DEMs were found between DAP versus CCI and CCI versus sham groups, mainly including glycerophospholipids, fatty acyls, steroids and steroid derivatives, and sphingolipids (Figure 8C,D).

**Figure 9:**
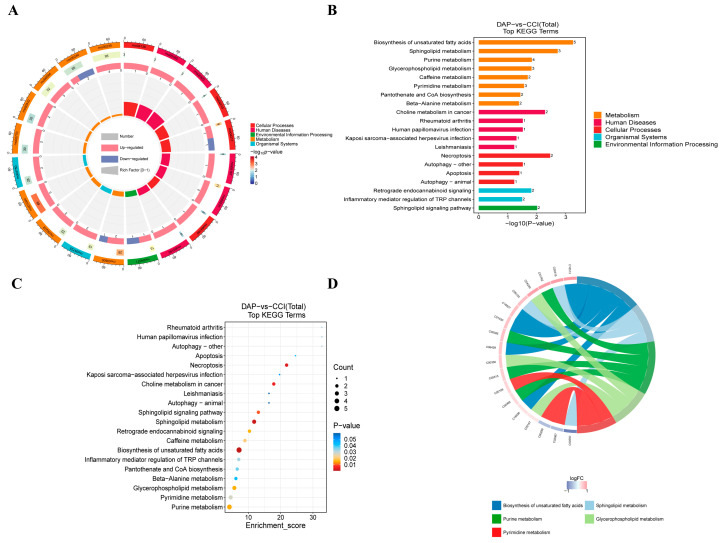
KEGG enrichment analysis of DEMs between the DAP and CCI groups. (**A**) Circos map of pathways. (**B**) Bar map of pathways. (**C**) Bubble map of pathways. (**D**) Chord map of pathways.

KEGG enrichment analysis showed that the DEMs between the DAP and CCI groups were mainly enriched in necroptosis, sphingolipid metabolism, retrograde endocannabinoid signaling, caffeine metabolism, biosynthesis of unsaturated fatty acid, glycerophospholipid metabolism, pyrimidine metabolism, and purine metabolism (Figure 9A–D).

### 2.7. Association Analysis of DEPs and DEMs between the DAP and CCI Groups

**Figure 10:**
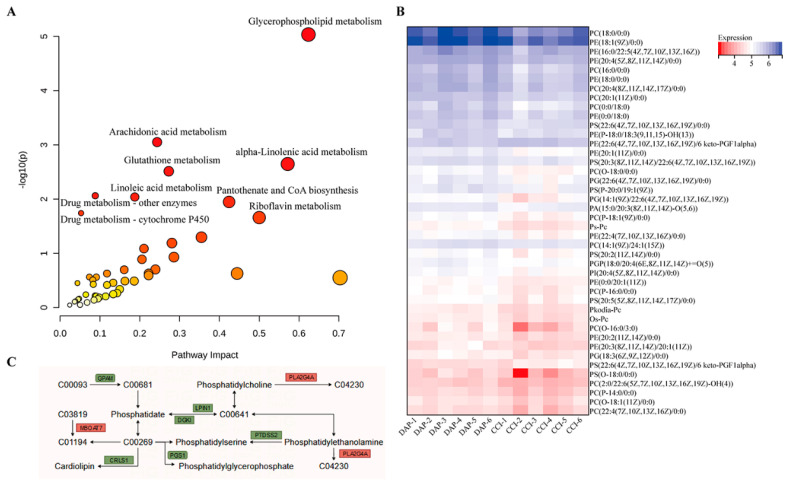
Association analysis of DEPs and DEMs between the DAP and CCI groups. (**A**) KEGG pathways. (**B**) Heat map of the 42 DEPs between the DAP and CCI groups. (**C**) Glycerophospholipid metabolism pathway.

To explore the relationships between proteins and metabolites, DEPs and DEMs were screened for further associations using MetaboAnalyst (https://www.metaboanalyst.ca, accessed on 29 April 2024). A total of nine metabolic pathways are shown in Figure 10A, namely, glycerophospholipid metabolism, arachidonic acid metabolism, alpha-linolenic acid metabolism, glutathione metabolism, drug metabolism—other enzymes, linoleic acid metabolism, pantothenate and CoA biosynthesis, drug metabolism—cytochrome P450, and riboflavin metabolism, with glycerophospholipid metabolism being the most significant. Further analysis revealed that DPA affected the synthesis of critical metabolites (phosphatidylserines (PSs), phosphatidylethanolamines (PEs), and phosphatidylcholines (PCs)) by regulating the expression of the lpin1, gpam, dgki, pla2g4a, ptdss2, pgs1, mboat7, and crls1 proteins (Figure 10B,C).

## 3. Discussion

The CCI model is comparatively stable and easy to replicate and involves neuropathy and inflammatory responses, which have been demonstrated in the relevant literature [28,29]. Therefore, we used the CCI model to explore the mechanism of DAP analgesia. In this study, we measured the MWT and TWT of CCI rats to evaluate allodynia in NP. CCI rats showed obvious mechanical and thermal allodynia that began on the first day after surgery and continued until the end of the experiment. Our data showed that intrathecal injection of DAP could significantly reduce NP caused by peripheral-nerve injury. Specifically, DAP effectively increased the TWT and MWT in model group rats, suggesting that DAP can inhibit mechanical and thermal pain sensitization after nerve injury.

It has been demonstrated through the lumbar radicular pain model that the activation of spinal microglia and the increase in IL-1β are involved in NP [30]. This study changed the perceived cause of NP from the activation of single neurons to a process involving glial cells and inflammatory factors. Under the effect of inflammatory factors, the release of electrical signals from neurons in the spinal cord increases, further exacerbating the sensation of pain [31]. In this study, we observed increased production of the IL-1β, IL-6, and TNF-α inflammatory factors in the spinal cord of the model group. After three consecutive days of intrathecal injection of DAP, it was found that the expression of the IL-1β, IL-6, and TNF-α proteins in the spinal cords of the DAPH group was significantly downregulated. This proved that DAP could effectively inhibit the expression of inflammatory factors in the spinal cord. Inhibition of inflammatory-factor production may be critical to the analgesic effect of DAP.

Microglia are immunocompetent cells in the central nervous system that are responsible for regulating the transmission of neuronal excitation [32]. Tsuda was the first to report the role of microglia in NP [33]. Purinergic P2 receptors, especially the ionotropic P2 subtype P2X_4_ receptors, were key receptors in the spinal cord microglia during the development of mechanical allodynia induced by peripheral nerve injury [33]. Interestingly, P2X_4_-receptor-induced pain hypersensitivity was sexually dimorphic in rats, and intrathecal injection of a P2X_4_ receptor antagonist attenuated CCI-induced pain hypersensitivity only in male rats [34]. It has been shown that P2X_4_ mRNA and P2X_4_ protein are not upregulated in the spinal microglia of females after neuropathy, in contrast to the effect in males, and that tactile pain is realized through a mechanism involving lymphocytes rather than microglial P2X_4_ [35,36]. We used male rats to investigate the modulation of microglial P2X_4_ receptors by DAP in the present study. However, a recent study showed that microglia P2X_4_ receptors are essential for both spinal neuron hyperexcitability and abnormal tactile pain in both male and female neuropathic mice [37]. This contradictory result may be due to differences in the breeding conditions of the mice tested. Because the environment may influence physiologic and pathologic responses in mice, it is critical to the pathogenesis of neuropathic pain [38]. After ligation of the rat sciatic nerve, we observed massive activation of microglia in the dorsal horn of the rat spinal cord and overexpression of P2X_4_ protein by the activated microglia. Intrathecal injection of DAP reversed this phenomenon. Based on the above findings, we speculate that DAP might inhibit microglial activation by interfering with P2X_4_ receptors.

Fibronectin in activated microglia regulates the translocation of IRF5 from the cytoplasm to the nucleus, and IRF5 induces the expression of the P2X_4_ receptors by directly binding to the promoter region of the P2X_4_ gene [39]. IRF8 is a critical regulator that selectively upregulates the expression of the P2X_4_ receptors by promoting IRF5 transcription in microglia [40]. Therefore, the IRF8/IFR5 transcription axis helps to regulate the activation of the P2X_4_ receptors of the ipsilateral spinal dorsal horn microglia after nerve injury. According to the western blot results, the expression of IRF8 and IRF5 proteins in the spinal cords of rats in the model group was significantly upregulated. Intrathecal injection of DAP could significantly inhibit the expression of IRF8 and IRF5 proteins. Therefore, we speculate that DAP regulates the P2X_4_ receptors through the IRF8/IRF5 axis. However, it is not yet clear how DAP affects upstream IRF8 protein expression. IRF8 transcription can be regulated by colony-stimulating factor 1 and a triggering receptor expressed on myeloid cell 2 proteins [41], and we are unsure whether DAP regulates IRF8 protein expression by modulating these signaling molecules. DAP may also be able to affect IRF8 protein stability. Future studies should explore the specific molecular mechanisms by which DAP regulates IRF8/IRF5/P2X_4_ and clarify the effective concentration of DAP in the spinal cord.

In the early stage of the experiment, it was found that DAP inhibited the activation of microglia in the spinal dorsal horn of CCI rats and the upregulation of inflammatory factors by interfering with P2X_4_ receptors. A variety of purinergic receptors can be used as initiators and regulators of the MAPK pathway and participate in the induction of pain sensitization [42]. P2X_4_ purinergic receptors can be used as the initiating factor and regulator of the MAPK pathway to induce pain sensitization [42]. Therefore, we hypothesize that DAP may interfere with the synthesis and release of inflammatory factors by regulating MAPK-pathway-related target proteins in microglia through P2X_4_. The activation of the MAPK pathway induces the expression of various inflammatory factors, such as TNF-α, IL-1β, IL-6, and prostaglandin E2 [43,44], a finding consistent with our previous WB experimental data. We found that the expression of inflammatory factors in the spinal cords of CCI rats was significantly reduced after intrathecal injection of DAP, which may be associated with DAP’s effect on the MAPK pathway in microglia in the spinal cords of CCI rats. P38 MAPK is activated by the upstream kinase MKK3/MMK6, which is considered a stress-induced kinase and plays a vital role in the inflammatory response [45]. Intrathecal injection of anti-inflammatory cytokines reduces spinal cord-injury-induced inflammation and inhibits spinal cord-injury-induced P38 MAPK activation [46]. To explore how DAP interferes with the MAPK pathway, we detected P38, JNK, and ERK1/2 and their phosphorylated proteins. In the experimental results, we found that p-P38/P38 was significantly decreased in the DAPH group after intrathecal injection of DAP. DAP did not affect all three MAPK pathways as envisaged, but significantly inhibited only p-P38/P38. These findings, combined with the results of double immunofluorescence staining, also confirmed the inhibitory effect of DAP on P38 MAPK in microglia. In summary, DAP reduces the expression of inflammatory factors in the spinal cord by inhibiting the P38 MAPK pathway in microglia.

BDNF is a neurotrophic factor with an important role in neuronal survival and differentiation. However, in addition to its classical neurotrophic effects, BDNF, as a neuromodulator, may be directly involved in the control of neuronal activity and synaptic plasticity through TrkB, increasing neuronal electrical excitability [47]. In addition, the activation of TrkB by BDNF reduces the expression of neuronal KCC2, thereby converting the normal inhibitory effect of GABA into an excitatory effect [48]. Peripheral nerve injury induces activation of P2X_4_ receptors in spinal microglia, which in turn promotes BDNF release and induces hyperexcitability of dorsal horn neurons, leading to central sensitization. In the present study, DAP suppressed BDNF expression by inhibiting P2X_4_ receptor activation in microglia, thereby inhibiting neuronal hyperactivation and thus exerting analgesic effects. Notably, microglia-derived BDNF was also found to play a key role in neuronal excitability and pain sensitization in supraspinal structures such as thalamic nuclei, cortical regions, and the midbrain ventral tegmental area [49]. Thus, microglia-derived BDNF may also contribute to chronic-pain-related complications (e.g., depression and anxiety). The modulation of the supraspinal structural BDNF pathway by DAP and its role in pain-related complications needs to be further explored in the future.

Finally, proteomics and metabolomics results showed that the CCI model could lead to disturbances in multiple metabolic pathways such as glycerophospholipid metabolism, glutathione metabolism, and arachidonic acid metabolism, which may contribute to NP by exacerbating neuroinflammation. We found that the glycerophospholipid metabolism pathway was the most significant. Glycerophospholipids are an essential class of lipids that constitute the majority of phospholipids in mammals, play a crucial role in biofilm formation, and are important elements in protein recognition and signal transduction [50]. Previous studies have shown that glycerophospholipid metabolism is involved in inflammatory states, and disorders of glycerophospholipid metabolism have been associated with the development of a variety of diseases such as rheumatoid arthritis, diabetes mellitus, and depression by inducing inflammatory responses and autoimmunity [51,52,53]. Glycerophospholipid metabolism is also associated with NP. The levels of PC (16:0/18:1) and PC (18:0/18:1) in the DRG were decreased in the sciatic-nerve-transection-induced NP mouse model, which may be related to the production of lysophosphatidic acid (LPA) [54]. Also, the levels of 16:0-, 18:0-, and 18:1-LPA in the spinal cord were increased in the partial-sciatic-nerve-ligation-induced NP mouse model [55]. LPA is associated with the development of inflammation, and some studies have shown that LPA antagonists attenuate NP in vivo [56,57]. Thus, disturbances in glycerophospholipid metabolism induced by peripheral nerve injury may induce neuroinflammation, further promoting central sensitization by promoting microglia activation and neuronal excitation. The PA (15:0/20:3(8Z,11Z,14Z)-O(5,6)) level was reduced in the CCI group in this study. Therefore, we speculate that PA (15:0/20:3(8Z,11Z,14Z)-O(5,6)) was consumed to produce LPA in the CCI group and that DAP treatment inhibited this process. LPA has been reported to activate transient receptor potential vanilloid 1 (TRPV1) and 4 (TRPV4) channels, and TRPV1 and TRPV4 channel activation play a key role in the generation and development of pathological pain perception [58]. Therefore, DAP may improve mechanical and thermal allodynia in CCI rats by inhibiting TRPV1 and TRPV4 channel activation through the modulation of glycerophospholipid metabolism. In addition, a recent study demonstrated that TRPV4 channels in microglia can mediate behaviors indicating neuropathic pain in a mouse model of spared nerve injury [59]. This suggests that DAP may exert analgesic effects by modulating glycerophospholipid metabolism and thus interfering with TRPV4 expression in microglia. PE and PC are key phospholipids of cell membranes and are involved in synthesizing the characteristic cellular bilayer structure [60]. PE (16:0/22:5 (4Z,7Z,10Z,13Z,16Z)) and PC (14:1(9Z)/24:1(15Z)) were significantly reduced in the CCI group in this study, suggesting altered phospholipid metabolism in the cell membranes and damage to the cell membranes; however, DAP treatment significantly reversed these changes. Further analysis showed that DAP could regulate the biosynthesis of PS, PE, and PC by upregulating the expression of the pla2g4a and mboat7 proteins and downregulating the expression of the lpin1, gpam, dgki, ptdss2, pgs1, and crls1 proteins.

Peripheral nerve injury induces oxidative damage caused by excessive ROS production in the spinal cord, leading to disruption of cell-membrane structure [61]. Activation of microglia, in addition to the release of inflammatory factors, leads to the overproduction of ROS in the spinal cord [62,63], thus disrupting neuronal biofilms and inducing disturbances in glycerophospholipid metabolism. The inhibition of oxidative stress may also be one of the mechanisms by which DAP exerts its analgesic effects. In conclusion, our results suggest that glycerophospholipid metabolism may be a metabolic feature of NP and that DAP can restore aberrant glycerophospholipid metabolism, which may contribute to the attenuation of neuroinflammation and central sensitization in the spinal cords of NP rats. However, the molecular mechanisms by which DAP regulates the above metabolic pathways are unknown. Further studies are necessary to confirm whether the metabolic imbalance induced by peripheral nerve injury, which also affects the metabolism of microglia and astrocytes, is limited to neuronal cells.

## 4. Materials and Methods

### 4.1. Animals

Six-week-old male Sprague–Dawley (SD) rats (*n* = 66) weighing 180–200 g were provided by SPF Beijing Biotechnology Co., Ltd. (No. SCXK2020-0033, Beijing, China). All rats were maintained with a 12 h light/12 h dark cycle at a constant temperature (23 ± 1 °C), and relative humidity (60% ± 5%) under standardized conditions, and food and water were freely available. All rats were allowed to adapt to the environment for a week before the experiment. The animal experimental protocol of this study was reviewed and approved by the Medical and Experimental Animal Ethics Committee of Beijing University of Chinese Medicine (No. BUCM4-2023022201-1012).

### 4.2. Establishment of Model and Drug Administration

After one week of adaptive feeding, the rats were anaesthetized with pentobarbital sodium (020402, Beijing Chemical Reagent Company, Beijing, China) (1.5%, i.p.) and underwent spinal cord subdural intubation. The lumbar muscles of the rats were separated, and the intervertebral foramen of the first caudal vertebra was fully exposed. The EP-10 catheter (302021, Smiths Medical, London, UK) (filled with saline) was inserted 1.5 cm forward from the level of the intervertebral foramen. The muscles and skin of the rats were sutured and sterilized, the catheter was closed, and the rats were fed in a single cage. Lidocaine hydrochloride (XB19H26, Hualu Pharmaceutical Co., Ltd., Liaocheng, China) was injected intrathecally on postoperative day 5, and the lower limbs of the rats were paralyzed immediately after the injection, indicating that the catheter was located at the L4−L5 position [64].

On the seventh day after the operation, all groups except for the control group and the sham group were subjected to the experimental conditions of the CCI model [65]. The rats were placed prone on the operating table, and the left hind limb was depilated and disinfected. The biceps femoris was separated, and the sciatic nerve trunk was exposed and ligated with a 4-0 surgical suture (SUS304, Jinhuan, Shanghai, China) four times, with a space of about 1 mm each time. After ligation, the muscles and skin were sutured and disinfected. In the sham group, only the sciatic nerve was exposed without ligation, and the rest of the operation was performed as above. The criteria for successful modeling were claudication, foot lifting and licking, thermal- and pain-threshold reduction, and pain sensitization in the affected limb.

In experiment 1, rats were divided into 6 groups (*n* = 6) in the pharmacodynamic study to analyze the mechanism of DAP activity: the control, sham, CCI, positive (3 mg/mL morphine) (850807, Qinghai pharmaceutical factory, Qinghai, China), DAP high-dose (DAPH; 0.5 mg/mL) (B21084, Yuanye Biotechnology Co., Ltd., Shanghai, China), and DAP low-dose (DAPL; 0.2 mg/mL) groups. In experiment 2, the rats were divided into 3 groups (*n* = 4 in proteomics and *n* = 6 in metabolomics) in the omics experiment for DAP: the sham, CCI, and DAPH groups. All drugs were dissolved in saline. The administration group was given 25 μL per injection twice daily for 3 days. The control, sham, and CCI groups were injected with the same amount of saline twice daily for 3 days.

### 4.3. Behavioral Tests

The MWT and TWT were measured using von Frey hair (NC12775, Yuyan Scientific Instrument Co., Ltd., Shanghai, China) and a cold/hot disk pain meter (PE34, IITC, Woodland Hills, CA, USA), respectively. In the MWT test, the rats were placed in a metal grid cage to allow them to adapt to the environment for 30 min. The tips of the von Frey hair were used to perpendicularly stimulate the mid-plantar surface of the rats’ left hind paws five times at five-minute intervals, and the amount associated with 50% positive reaction was calculated (ED50) [66]. In the TWT test, after the rats had been acclimatized to the environment for 5 min, the instrument was activated, and the temperature was observed and recorded when the rats licked their toes. Each rat was measured three times at intervals of 10 min. The average value was calculated for the TWT [67].

### 4.4. Immunofluorescence Staining

After perfusion fixation, the muscles and vertebrae were separated and the spinal cords of L4−L5 rats were removed. Spinal cord tissue was fixed in 10% neutral formalin, dehydrated with ethanol, and embedded in wax. The slice thickness was 4 µm. Spinal cord sections were incubated with Antibody-Iba-1 (1:200, ab178846, Abcam, Cambridge, UK), Antibody-P2X_4_ (1:200, bs-7690R, Bioss, Beijing, China), Antibody-c-fos (1:100, ab208942, Abcam, Cambridge, UK), and Antibody-P38 (1:50, 14064-1-AP, Proteintech, Wuhan, China) at 4 °C overnight. Goat anti-rabbit IgG H&L (Alexa Flour 488, 1:200, ab150077, Abcam, Cambridge, UK) and goat anti-mouse IgG H&L (Alexa Flour 647, 1:200, ab150115, Abcam, Cambridge, UK) were selected as secondary antibodies. After immunofluorescence staining, the tissue sections were observed and photographed under a laser confocal microscope (Sp8, Leica, Wetzlar, Germany). Quantitative fluorescence analysis was performed using Image J 1.53.

### 4.5. Proteomics Analysis

Proteomics analysis was performed using mass spectrometry in data-dependent acquisition mode. FC > 1.2 or < 1/1.2 and *p* < 0.05 using Student’s *t*-test were set as cut-off values to identify significant changes in protein expression. The DEPs were mapped to the KEGG database (https://www.kegg.jp/kegg/pathway.html, accessed on 29 April 2024) to identify enriched KEGG pathways.

### 4.6. Untargeted Metabolomic Analysis

Untargeted metabolomics analysis of the sham, CCI, and DAP groups was performed using LC-MS/MS. The LC-MS/MS analysis was conducted on a quadrupole electrostatic field orbitrap high-resolution mass spectrometer (Thermo Fisher Scientific, USA). Assignment of metabolites was determined based on the published data and publicly available databases such as HMDB (https://hmdb.ca/, accessed on 29 April 2024), KEGG (http://www.genome.jp/kegg, accessed on 29 April 2024), PubChem compound database (http://pubchem.ncbi.nlm.nih.gov, accessed on 29 April 2024), and Lipid Maps (https://www.lipidmaps.org/, accessed on 29 April 2024). The processed data were subjected to multivariate and univariate data analysis, including PCA, OPLS-DA, FC analysis, Student’s *t*-test, and KEGG enrichment analysis. The filtering criteria of DEMs were defined as VIP > 1 and *p* < 0.05.

### 4.7. Western Blot Analysis

L4−L5 spinal cords of 3 rats selected from each group were homogenized in RIPA lysate buffer (P0013C, Beyotime, Haimen, China) supplemented with protease and phosphatase inhibitor (20210401, NCM, Tianjin, China). The protein concentration was determined using a BCA protein-detection kit (120320210419, Beyotime, Haimen, China). Total protein (10 μg) was separated by means of SDS-PAGE and electrically transferred to the PVDF membrane (R1DB92455, Millipore, Darmstadt, Germany). The PVDF membranes containing the proteins were blocked for 1.5 h and incubated with the primary antibody overnight at 4 °C. The primary antibodies were as follows: Antibody-IL-1β (1:1000, ab254360, Abcam, Cambridge, UK), Antibody-IL-6 (1:1000, ab259431, Abcam, Cambridge, UK), Antibody-TNF-α (1:1000, ab183218, Abcam, Cambridge, UK), Antibody-α-Tubulin (1:5000, ab7291, Abcam, Cambridge, UK), Antibody-P2X_4_ (1:1000, bs-7690R, Bioss, Beijing, China), Antibody-BDNF (1:1000, 66292-1-Ig, Proteintech, Wuhan, China), Antibody-IRF8 (1:1000, 18977-1-AP, Proteintech, Wuhan, China), Antibody-IRF5 (1:1000, ab181553, Abcam, Cambridge, UK), Antibody-β-actin (1:5000, ab8226, Abcam), Antibody-P38 (1:1000, 14064-1-AP, Proteintech, Wuhan, China), Antibody-p-P38 (1:1000, 4511S, CST, Boston, MA, USA), Antibody-JNK (1:5000, 66210-1-Ig, Proteintech, Wuhan, China), Antibody-p-JNK (1:2000, 9255S, CST, Boston, MA, USA), Antibody-ERK1/2 (1:5000, 67170-1-Ig, Proteintech, Wuhan, China), and Antibody-p-ERK1/2 (1:2000, 4370S, CST, Boston, MA, USA). Then, the membranes were washed three times and incubated with Goat anti-rabbit IgG HRP (1:5000, S0101, LABLEAD, Beijing, China) or Goat anti-mouse IgG HRP (1:5000, S0100, LABLEAD, Beijing, China) for 1 h at room temperature. The protein-band signal was detected with the Amersham ECL system (Amersham imager 680, Pittsburgh, PA, USA). The protein bands were analyzed quantitatively using Image J 1.53 software.

### 4.8. Statistical Analysis 

SAS 8.2 was used for statistical processing, and all the data are expressed as the mean ± standard deviation (S.D.). When the sample size did not conform to a normal distribution, a nonparametric test was used. When the data conformed to the normal distribution, a two-way Student’s *t*-test was used for comparison between two groups (unpaired), one-way ANOVA was used for three or more groups, and the LSD method was used to compare the groups. Behavioral data were analyzed using the repeated-measures ANOVA method. The threshold for significance was set to *p* < 0.05.

## 5. Conclusions

In conclusion, DAP alleviates NP by inhibiting the P2X_4_/P38 pathway to suppress microglial responses and by ameliorating imbalances in glycerophospholipid metabolism. This study reveals NP’s metabolic characteristics and a potential mechanism by which DAP treats NP.

## Data Availability

Data are contained within the article.
